# Sleeping with the Enemy: How Intracellular Pathogens Cope with a Macrophage Lifestyle

**DOI:** 10.1371/journal.ppat.1002551

**Published:** 2012-03-22

**Authors:** Emily P. Thi, Ulrike Lambertz, Neil E. Reiner

**Affiliations:** 1 Department of Medicine (Division of Infectious Diseases), University of British Columbia, Faculties of Medicine and Science, and Vancouver Coastal Health Research Institute (VCHRI), Vancouver, British Columbia, Canada; 2 Department of Microbiology and Immunology (Division of Infectious Diseases), University of British Columbia, Faculties of Medicine and Science, and Vancouver Coastal Health Research Institute (VCHRI), Vancouver, British Columbia, Canada; University of Wisconsin Medical School, United States of America

## Introduction

Intracellular pathogens are a major cause of global morbidity and mortality. While this alone establishes their medical importance, they are also a focus of special interest because of their unique lifestyles. Many of these organisms have evolved to reside within the hostile environment of macrophages. Given that these innate immune effector cells are normally programmed to destroy ingested prey and promote the development of adaptive immunity, this is one of the ultimate paradoxes in the study of host–pathogen interactions. The success of these microbes is dependent on diverse strategies including the disruption of macrophage cell regulation, the ability to nullify macrophage microbicidal effector mechanisms, and other special adaptations to an intracellular lifestyle. Here, we review a series of well established survival paradigms that have emerged that illustrate this behaviour.

## Targeting Critical Signaling Pathways Prevents Effective Macrophage Activation and Promotes Survival of Intracellular Pathogens

Activated macrophages form a heterogeneous collection of cells with functions as diverse as secretion of pro- or anti-inflammatory mediators, intracellular pathogen killing, induction of T-helper 1 or T-helper 2 cell responses, extracellular matrix synthesis and tissue repair, and others. Not surprisingly, many intracellular microbes interfere with host cell signaling in order to prevent or modulate cellular activation in their favour.

One notable example involves interference with host protein kinase and phosphatase activities, thereby preventing macrophage activation. *Leishmania* release effectors that activate macrophage phosphotyrosine phosphatases, leading to inhibition of Jak-STAT and mitogen-activated protein (MAP) kinase signaling [1] (and see also supplemental references [S1,S2,S3] in Text S1). The *Leishmania* surface lipophosphoglycan (LPG) [2] and *Mycobacterium tuberculosis* (Mtb) protein kinase G [3] both suppress protein kinase C activity, thereby inhibiting the macrophage oxidative burst in response to *Leishmania* [S4] and attenuating phagocytosis of Mtb [3]. Activation of PI3K has been observed during infection with Mtb [S5], *Francisella tularensis* [4], and *Leishmania* [5,S6], leading to inhibition of both apoptosis and pro-inflammatory cytokine secretion with reciprocal enhancement of anti-inflammatory cytokine production. Furthermore, pathogens such as *Leishmania amazonensis* and Mtb modulate host protein synthesis through effects on protein kinase R, or interference with Toll-like receptor (TLR)-mediated activation of the transcription factor NFκB [S7,S8].

Intracellular microbes can shape macrophage phenotypes by modulating cytokine secretion, which determines the fate of the downstream host response. Most pathogens inhibit production of pro-inflammatory cytokines such as IL-12, TNF-α, and IFN-γ, and induce production of anti-inflammatory cytokines such as IL-10, TGF-β, and IL-4, thereby down-regulating immunopathology and host defence mechanisms. Importantly, signaling pathways centered on IFN-γ, the predominant inducer of macrophage-mediated microbicidal functions, are commonly targeted by intracellular pathogens. Down-regulation of IFN-γ receptor expression or interference with the IFN-γ receptor downstream effectors Jak and STAT have been observed during infection with Mtb [S9,S10], *Trypanosoma cruzi* [S11], and *Leishmania* [S2,S12].

Prolonged survival in macrophages also requires prevention of adaptive immunity. To this end, Mtb inhibits expression of the class II transactivator and MHC class II molecules and antigen presentation, thereby evading effective recognition by CD4^+^ T cells (for a review see [6]). Similar phenomena have been observed during infection with *Toxoplasma gondii* [S13], *Leishmania* [S14,S15], and *Salmonella* [S16].

## Coping with an Intracellular Lifestyle May Involve Escaping from the Phagosome, Modulating Phagosome Maturation, or Liaisons with Autophagosomes

Phagocytic uptake of pathogens by macrophages results in the formation of vacuoles that, by interactions with vesicles of the endosomal system, are intended to transform into microbicidal phagolysosomes through a process known as phagosome maturation. Intracellular pathogens residing within phagosomes, however, have evolved mechanisms to either subvert or delay the maturation process, or to escape from their vacuoles altogether.

Organisms such as Mtb and *Salmonella* block maturation by targeting key regulatory pathways including PI 3-kinase signalling and small GTPase Rab conversions, which ordinarily promote the acquisition and activation of effectors needed for maturation to occur. Both Mtb [7,S17,S18] and *Salmonella enterica typhimurium* [S19,S20] secrete phosphoinositide phosphatases that dephosphorylate phosphoinositides on the phagosome membrane. Mtb [S21–S25] and *S. typhimurium* [S26,S27] also actively prevent the acquisition and activation of Rab7 and its downstream effectors. Similarly, *S. typhimurium* interferes with Rab9 recruitment to the *Salmonella*-containing vacuole [S28]. In addition, Mtb secretes protein tyrosine phosphatase A, which interferes with late endosome/lysosome vesicular interactions [8], and its cell wall lipid ManLAM modulates MAP kinase signaling, leading to phagosome maturation arrest between early and late stages [S29,S30].

Other pathogens such as *Legionella pneumophila* [S31,S32], *Leishmania* [S33–S35], and *Coxiella burnetti* [S36–S38] do not arrest, but rather delay, phagosome maturation in order to provide time to switch from an extracellular form to one that is able to cope with conditions in a phagolysosome [9]. *Leishmania* delay maturation using their surface-associated lipophosphoglycan, during which time these protozoan parasites switch from the promastigote form to the phagolysosome-resistant amastigote form [S39]. *Legionella* [S40] and *Coxiella* secrete effectors via their Type IV secretion system that aid in delaying phagosome maturation. In addition, *Legionella* surface composition and the secretion of microvesicles delay maturation independent of Type IV secretion [S41].

Interestingly, intracellular pathogens not only adapt to resist the harsh conditions in the phagolysosome, but may actually require lysosomal as well as autophagosomal interactions to facilitate the delivery of nutrients [10,S42–S45]. Autophagy is a mechanism whereby cells recycle cellular components by enclosing them in autophagosomes that fuse with lysosomes to mediate degradation of their contents. Although autophagy plays a role in host defence by encapsulating and degrading microbes, this pathway is also important in facilitating survival of *Chlamydia* [S46], *Brucella abortus* [S47], and *Yersinia* [S48,S49].

Not all pathogens that enter the host cell in a phagosome remain there. *Listeria monocytogenes* [S50,S51] and *Shigella flexneri* [S52,S53] escape from phagosomes by forming actin tails. This enables them to move within the infected cell, preventing their recognition and killing by autophagy and allowing dissemination to neighbouring cells [11]. Unlike *L. monocytogenes* and *S. flexneri*, which leave their phagosomes at an early stage of phagosome maturation, the protozoan *T. cruzi* escapes from its vacuole after lysosomal fusion, when low pH conditions favour hemolysin activity [S54,S55].

## Acquisition of Essential Nutrients and Co-Factors from Host Cells Promotes Growth and Replication of Intracellular Pathogens

In order to survive and replicate within their host cells, microbes must gain access to nutrients. Pathogens such as *L. monocytogenes* and *S. flexnerii* escape from phagosomes into the cytosol, which is relatively nutrient-rich. In contrast, persistent phagosome dwellers face restrictive conditions for access to metal cations such as Fe^2+^, Mn^2+^, and Co^2+^, as these are transported out of the phagosome via the Nramp (natural resistance–associated macrophage protein) transporter [12,S56]. To compete against Nramp, certain bacteria express an Nramp homolog [S57,S58]. This allows them to acquire cation cofactors that are essential for bacterial reactive oxygen intermediate (ROI) detoxifying enzymes [13]. Since greater than 80% of total body iron is bound to heme, and iron is further sequestered by transferrin and lactoferrin, free iron is in short supply in host cells. This serves as an important mechanism of resistance against microbial invaders. To obtain iron, *Leishmania* express receptors that allow internalisation of hemoglobin, transferrin, and lactoferrin [14,S59,S60], and exploit the iron labile pool in macrophages [S61]. Bacterial pathogens express iron-chelating siderophores such as mycobacterial mycobactins [15], salmochelin released by *Salmonella* and *Shigella* [S62,S63], and *Yersinia* yersiniabactin [S64], all of which compete with transferrin or lactoferrin for iron. In conjunction with siderophore production, Mtb also express a heme uptake system [S65].

In addition to iron, pathogens whose niche is restricted to phagosomes must also acquire carbon. Mtb uses fatty acids and cholesterol as a carbon source and this is essential for persistence [16,S66,S67]. *Salmonella* uses host glucose, phosphorylated carbohydrates, and fatty acids as carbon sources [S68,S69]. Pathogens such as *Leishmania* [S42], *Chlamydia* [S46], and *Coxiella* [S44,S70,S43] also reside in phagolysosomes that intersect with autophagosomes that provide amino acids and other nutrients.

## Strategies Used to Prevent or Resist the Phagocyte Oxidative Burst or the Formation of Reactive Nitrogen Intermediates

A major weapon in the macrophage antimicrobial arsenal is the phagocyte oxidative burst, which results in the generation of ROIs such as superoxide, hydrogen peroxide, and hypochlorous acid. The importance of the oxidative burst to host defence is illustrated by patients with chronic granulomatous disease who, due to oxidase mutations, cannot form ROIs and consequently suffer from recurrent bacterial and fungal diseases.

There are at least two strategies by which intracellular pathogens modulate the oxidative burst: (1) by preventing assembly of the macrophage NADPH oxidase that produces ROIs, or (2) by detoxifying ROIs as they are formed. The *Salmonella* pathogenicity island (SPI)-2, which encodes for a Type III secretion apparatus, is necessary for preventing NADPH oxidase assembly on the phagosome membrane [17,S71]. *Leishmania* also prevent NADPH oxidase assembly through surface LPG, which serves a dual role by also scavenging ROIs directly [S72,S35].

Apart from targeting NADPH assembly and activation, many pathogens express enzymes that neutralize ROIs. *L. monocytogenes* [S73], *Salmonella* [18], *Yersinia* [S74], *F. tularensis* [S75], and Mtb [S76,S77] all express surface-associated and cytoplasmic superoxide dismutases, which scavenge superoxide radicals. In addition, many of these pathogens also secrete catalase-peroxidase, which breaks down H_2_O_2_ [S78–S82].

The catalase-peroxidases of *Salmonella* and Mtb can also act as peroxynitritases that deactivate peroxynitrite formed when nitric oxide (produced by inducible nitric oxide synthase [iNOS]) reacts with superoxide [19,S80]. *T. cruzi* also deactivates peroxynitrite, which is critical for pathogen survival within the host macrophage [S83]. The expression of iNOS and production of reactive nitrogen intermediates (RNIs) is a critical macrophage microbicidal mechanism dependent upon the oxidation of L-arginine. iNOS activity competes for this amino acid with arginase 1, which promotes the production of urea. Mtb skews the balance by upregulating arginase expression via autocrine cytokine signaling in infected macrophages in order to curtail iNOS activity [20]. If RNIs are indeed formed, certain microorganisms have detoxification mechanisms to call upon, such as *Salmonella* factors secreted by Type III secretion [21], and the truncated hemoglobins of Mtb [S84], *Mycobacterium bovis* [S85], and *Mycobacterium leprae* [S86]. In parallel with detoxification of RNIs, *Salmonella* also repair nitrosative damage using flavohemoglobins [22], flavorubredoxin, and cytochrome C nitrite reductase [S87].

## Manipulation of Macrophage Death Pathways Supports an Intracellular Lifestyle

When macrophages fail to eliminate infecting pathogens, programmed cell death is often initiated as a last resort to resolve the infection. Three major modes of cell death may occur in response to infection: apoptosis, necrosis, and pyroptosis. Apoptosis is characterised by retention of membrane integrity coupled with the formation of apoptotic bodies and is generally viewed as being immunologically silent. Apoptotic cells are engulfed by neighbouring phagocytes, which may result in pathogen degradation and initiation of adaptive immunity. In contrast, necrosis and pyroptosis trigger rapid inflammatory responses, as necrotic and pyroptotic cells secrete pro-inflammatory cytokines and release cytoplasmic contents extracellularly.

In order to retain their intracellular niche, many intracellular pathogens prevent host cell death. A major mechanism to inhibit apoptosis is interference with caspase signaling cascades. Inhibition of effector caspases 3 and 8 has been observed during infection with *T. gondii* [S88] and *T. cruzi* [S89]. Activation of caspase death cascades can also be subverted by preventing cytochrome C release from mitochondria, a strategy shared by *T. gondii* [S90] and *Leishmania* [S91]. Other pro-survival, host signaling cascades exploited by pathogens to confer resistance to apoptosis are activation of the PI3K/Akt axis by *L. major* and *L. amazonensis* [5] and *T. gondii* [23] and activation of NFκB by *Ehrlichia chaffeensis* [S92], *L. monocytogenes* [S93], and *L. pneumophila* [S94,S95]. Activation of these pathways results in the upregulation of pro-survival molecules of the Bcl-2 family, inhibition of cytochrome C release, and activation of inhibitor of apoptosis proteins (IAPs). Additional strategies to inhibit apoptosis include expression of orthologs of host anti-apoptotic molecules, such as macrophage migration inhibitory factor by *L. major* [S96], or induction of anti-apoptotic heat shock proteins by *T. gondii* [S97]. Prevention of pyroptotic cell death has been demonstrated for *Yersinia enterocolitica* and Mtb, brought about by inhibition of caspase-1 activation and inflammasome formation [24,S98].

Conversely, several pathogens induce cell death in order to promote dissemination, or to evade systemic immunity or both. Whereas intracellular pathogens generally avoid initiating pro-inflammatory necrosis or pyroptosis, initiation of macrophage apoptosis may benefit the pathogen by promoting dissemination to other macrophages without activating them and often reflects a productive infectious cycle. Initiation of macrophage apoptosis has been reported for *T. cruzi* [25], *L. major*, *Leishmania aethiopica*, *and Leishmania tropica* [26], and *Yersinia pseudotuberculosis* [27,S99]. The fact that both *T. cruzi* and *Leishmania* spp. have been shown to be capable of either preventing or inducing apoptosis at first blush appears paradoxical. It seems plausible, however, that these juxtaposed behaviours may reflect different stages of infection. Thus, prevention of apoptosis early after infection may allow for preservation of the intracellular niche and replication, whereas during late stage infection, induction of apoptosis facilitates pathogen release, dissemination, and silent uptake by other macrophages. This behaviour is also exhibited by virulent Mtb, which has been shown to prevent apoptosis, but induce necrosis of infected macrophages to promote the spread of bacilli [28,S100]. It has further been shown that *T. gondii* [S101], *Leishmania donovani* [S102], and *M. bovis* [S103] induce apoptosis of non-infected bystander cells to deplete monocytes, macrophages, and CD4^+^ and/or CD8^+^ T cells.

## Concluding Remarks

Intracellular pathogens have evolved a wide and diverse range of strategies to avoid or defend against macrophage innate immune effector mechanisms. In response, macrophages are equipped with various countermeasures such as phosphorylating secreted microbial superoxide dismutases to inactivate them [29] and the production of host proteins that recognize and bind to microbial iron chelators, thereby preventing pathogen iron uptake [30,S104]. A deeper understanding of these macrophage countermeasures and their regulation, as well as how pathogens regulate their own responses to the macrophage environment, will prove to be fertile ground for developing targeted therapeutic strategies in years to come.

## Supporting Information

Text S1 Supplementary references are cited throughout as “S1, S2…” and are listed in Text S1.
(DOC)

## References

[1] Nandan D, Yi T, Lopez M, Lai C, Reiner NE (2002). *Leishmania* EF-1alpha activates the Src homology 2 domain containing tyrosine phosphatase SHP-1 leading to macrophage deactivation.. J Biol Chem.

[2] Descoteaux A, Matlashewski G, Turco SJ (1992). Inhibition of macrophage protein kinase C-mediated protein phosphorylation by *Leishmania donovani* lipophosphoglycan.. J Immunol.

[3] Chaurasiya SK, Srivastava KK (2009). Downregulation of protein kinase C-alpha enhances intracellular survival of Mycobacteria: role of PknG.. BMC Microbiol.

[4] Medina EA, Morris IR, Berton MT (2010). Phosphatidylinositol 3-kinase activation attenuates the TLR2-mediated macrophage proinflammatory cytokine response to *Francisella tularensis* live vaccine strain.. J Immunol.

[5] Ruhland A, Leal N, Kima PE (2007). *Leishmania* promastigotes activate PI3K/Akt signalling to confer host cell resistance to apoptosis.. Cell Microbiol.

[6] Harding CV, Boom WH (2010). Regulation of antigen presentation by *Mycobacterium tuberculosis*: a role for Toll-like receptors.. Nat Rev Microbiol.

[7] Vergne I, Chua J, Lee HH, Lucas M, Belisle J (2005). Mechanism of phagolysosome biogenesis block by viable *Mycobacterium tuberculosis*.. Proc Natl Acad Sci U S A.

[8] Bach H, Papavinasasundaram KG, Wong D, Hmama Z, Av-Gay Y (2008). *Mycobacterium tuberculosis* virulence is mediated by PtpA dephosphorylation of human vacuolar protein sorting 33B.. Cell Host Microbe.

[9] Swanson MS, Fernandez-Moreira E (2002). A microbial strategy to multiply in macrophages: the pregnant pause.. Traffic.

[10] Alexander J, Vickerman K (1975). Fusion of host cell secondary lysosomes with the parasitophorous vacuoles of *Leishmania mexicana*-infected macrophages.. J Protozool.

[11] Yoshikawa Y, Ogawa M, Hain T, Yoshida M, Fukumatsu M (2009). *Listeria monocytogenes* ActA-mediated escape from autophagic recognition.. Nat Cell Biol.

[12] Vidal SM, Malo D, Vogan K, Skamene E, Gros P (1993). Natural resistance to infection with intracellular parasites: isolation of a candidate for Bcg.. Cell.

[13] Agranoff D, Monahan IM, Mangan JA, Butcher PD, Krishna S (1999). *Mycobacterium tuberculosis* expresses a novel pH-dependent divalent cation transporter belonging to the Nramp family.. J Exp Med.

[14] Voyiatzaki CS, Soteriadou KP (1992). Identification and isolation of the *Leishmania* transferrin receptor.. J Biol Chem.

[15] De Voss JJ, Rutter K, Schroeder BG, Su H, Zhu Y (2000). The salicylate-derived mycobactin siderophores of *Mycobacterium tuberculosis* are essential for growth in macrophages.. Proc Natl Acad Sci U S A.

[16] McKinney JD, Honer zu BK, Munoz-Elias EJ, Miczak A, Chen B (2000). Persistence of *Mycobacterium tuberculosis* in macrophages and mice requires the glyoxylate shunt enzyme isocitrate lyase.. Nature.

[17] Vazquez-Torres A, Xu Y, Jones-Carson J, Holden DW, Lucia SM (2000). Salmonella pathogenicity island 2-dependent evasion of the phagocyte NADPH oxidase.. Science.

[18] Sly LM, Guiney DG, Reiner NE (2002). *Salmonella enterica* serovar Typhimurium periplasmic superoxide dismutases SodCI and SodCII are required for protection against the phagocyte oxidative burst.. Infect Immun.

[19] Wengenack NL, Jensen MP, Rusnak F, Stern MK (1999). *Mycobacterium tuberculosis* KatG is a peroxynitritase.. Biochem Biophys Res Commun.

[20] Qualls JE, Neale G, Smith AM, Koo MS, DeFreitas AA (2010). Arginine usage in mycobacteria-infected macrophages depends on autocrine-paracrine cytokine signaling.. Sci Signal.

[21] Chakravortty D, Hansen-Wester I, Hensel M (2002). *Salmonella* pathogenicity island 2 mediates protection of intracellular *Salmonella* from reactive nitrogen intermediates.. J Exp Med.

[22] Crawford MJ, Goldberg DE (1998). Role for the *Salmonella* flavohemoglobin in protection from nitric oxide.. J Biol Chem.

[23] Kim L, Denkers EY (2006). *Toxoplasma gondii* triggers Gi-dependent PI 3-kinase signaling required for inhibition of host cell apoptosis.. J Cell Sci.

[24] Master SS, Rampini SK, Davis AS, Keller C, Ehlers S (2008). *Mycobacterium tuberculosis* prevents inflammasome activation.. Cell Host Microbe.

[25] Freire-de-Lima CG, Nunes MP, Corte-Real S, Soares MP, Previato JO (1998). Proapoptotic activity of a *Trypanosoma cruzi* ceramide-containing glycolipid turned on in host macrophages by IFN-gamma.. J Immunol.

[26] Getti GT, Cheke RA, Humber DP (2008). Induction of apoptosis in host cells: a survival mechanism for *Leishmania* parasites?. Parasitology.

[27] Monack DM, Mecsas J, Ghori N, Falkow S (1997). *Yersinia* signals macrophages to undergo apoptosis and YopJ is necessary for this cell death.. Proc Natl Acad Sci U S A.

[28] Chen M, Gan H, Remold HG (2006). A mechanism of virulence: virulent *Mycobacterium tuberculosis* strain H37Rv, but not attenuated H37Ra, causes significant mitochondrial inner membrane disruption in macrophages leading to necrosis.. J Immunol.

[29] Archambaud C, Nahori MA, Pizarro-Cerda J, Cossart P, Dussurget O (2006). Control of *Listeria* superoxide dismutase by phosphorylation.. J Biol Chem.

[30] Johnson EE, Srikanth CV, Sandgren A, Harrington L, Trebicka E (2010). Siderocalin inhibits the intracellular replication of *Mycobacterium tuberculosis* in macrophages.. FEMS Immunol Med Microbiol.

