# Supplementary material for: Sleeping with the Enemy: How Intracellular Pathogens Cope with a Macrophage Lifestyle
Source: PLoS Pathog. 2012 Mar 22;8(3):e1002551. doi: 10.1371/journal.ppat.1002551 (PMC3310772; doi:10.1371/journal.ppat.1002551)
Supplement: Text S1 — Supplementary references are cited throughout as “S1, S2…” and are listed in Text S1. (DOC) [file ppat.1002551.s001.doc]

**Supplementary References**

S1. Gomez MA, Contreras I, Halle M, Tremblay ML, McMaster RW, Olivier M (2009) *Leishmania* GP63 alters host signaling through cleavage-activated protein tyrosine phosphatases. Sci Signal 2: ra58.

S2. Nandan D, Reiner NE (1995) Attenuation of gamma interferon-induced tyrosine phosphorylation in mononuclear phagocytes infected with *Leishmania donovani*: selective inhibition of signaling through Janus kinases and Stat1. Infect Immun 63: 4495-4500.

S3. Nandan D, Lo R, Reiner NE (1999) Activation of phosphotyrosine phosphatase activity attenuates mitogen-activated protein kinase signaling and inhibits c-FOS and nitric oxide synthase expression in macrophages infected with *Leishmania donovani*. Infect Immun 67: 4055-4063.

S4. Olivier M, Brownsey RW, Reiner NE (1992) Defective stimulus-response coupling in human monocytes infected with *Leishmania donovani* is associated with altered activation and translocation of protein kinase C. Proc Natl Acad Sci U S A 89: 7481-7485.

S5. Yang CS, Lee JS, Jung SB, Oh JH, Song CH, Kim HJ, Park JK, Paik TH, Jo EK (2006) Differential regulation of interleukin-12 and tumour necrosis factor - alpha by phosphatidylinositol 3-kinase and ERK 1/2 pathways during *Mycobacterium tuberculosis* infection. Clin Exp Immunol 143: 150-160.

S6. Nandan D, de Oliveira C, Moeenrezakhanlou M, Lopez M, Silverman J et al. (2011) Myeloid Cell Interleukin-10 Production in Response to *Leishmania* Requires Inactivation of Glycogen Synthase Kinase-3β Downstream of Phosphatidylinositol-3 Kinase. J Immunol. In press.

S7. Pathak SK, Basu S, Bhattacharyya A, Pathak S, Kundu M, Basu J (2005) *Mycobacterium tuberculosis* lipoarabinomannan-mediated IRAK-M induction negatively regulates Toll-like receptor-dependent interleukin-12 p40 production in macrophages. J Biol Chem 280: 42794-42800.

S8. Pereira RM, Teixeira KL, Barreto-de-Souza V, Calegari-Silva TC, De-Melo LD, Soares DC, Bou-Habib DC, Silva AM, Saraiva EM, Lopes UG (2010) Novel role for the double-stranded RNA-activated protein kinase PKR: modulation of macrophage infection by the protozoan parasite *Leishmania*. FASEB J 24: 617-626.

S9. Singhal A, Jaiswal A, Arora VK, Prasad HK (2007) Modulation of gamma interferon receptor 1 by *Mycobacterium tuberculosis*: a potential immune response evasive mechanism. Infect Immun 75: 2500-2510.

S10. Ting LM, Kim AC, Cattamanchi A, Ernst JD (1999) *Mycobacterium tuberculosis* inhibits IFN-gamma transcriptional responses without inhibiting activation of STAT1. J Immunol 163: 3898-3906.

S11. Kierszenbaum F, Mejia LH, Tanner MK, Sztein MB (1995) *Trypanosoma cruzi*-induced decrease in the level of interferon-gamma receptor expression by resting and activated human blood lymphocytes. Parasite Immunol 17: 207-214.

S12. Ray M, Gam AA, Boykins RA, Kenney RT (2000) Inhibition of interferon-gamma signaling by *Leishmania donovani*. J Infect Dis 181: 1121-1128.

S13. Luder CG, Walter W, Beuerle B, Maeurer MJ, Gross U (2001) *Toxoplasma gondii* down-regulates MHC class II gene expression and antigen presentation by murine macrophages via interference with nuclear translocation of STAT1α. Eur J Immunol 31: 1475-1484.

S14. Kima PE, Soong L, Chicharro C, Ruddle NH, Mahon-Pratt D (1996) Leishmania-infected macrophages sequester endogenously synthesized parasite antigens from presentation to CD4+ T cells. Eur J Immunol 26: 3163-3169.

S15. Reiner NE, Ng W, McMaster WR (1987) Parasite-accessory cell interactions in murine leishmaniasis. II. *Leishmania donovani* suppresses macrophage expression of class I and class II major histocompatibility complex gene products. J Immunol 138: 1926-1932.

S16. Mitchell EK, Mastroeni P, Kelly AP, Trowsdale J (2004) Inhibition of cell surface MHC class II expression by Salmonella. Eur J Immunol 34: 2559-2567.

S17. Beresford N, Patel S, Armstrong J, Szoor B, Fordham-Skelton AP, Tabernero L (2007) MptpB, a virulence factor from *Mycobacterium tuberculosis*, exhibits triple-specificity phosphatase activity. Biochem J 406: 13-18.

S18. Saleh MT, Belisle JT (2000) Secretion of an acid phosphatase (SapM) by *Mycobacterium tuberculosis* that is similar to eukaryotic acid phosphatases. J Bacteriol 182: 6850-6853.

S19. Bakowski MA, Braun V, Lam GY, Yeung T, Heo WD, Meyer T, Finlay BB, Grinstein S, Brumell JH (2010) The phosphoinositide phosphatase SopB manipulates membrane surface charge and trafficking of the Salmonella-containing vacuole. Cell Host Microbe 7: 453-462.

S20. Hernandez LD, Hueffer K, Wenk MR, Galan JE (2004) Salmonella modulates vesicular traffic by altering phosphoinositide metabolism. Science 304: 1805-1807.

S21. Seto S, Tsujimura K, Koide Y (2011) Rab GTPases regulating phagosome maturation are differentially recruited to mycobacterial phagosomes. Traffic 12: 407-420.

S22. Seto S, Matsumoto S, Tsujimura K, Koide Y (2010) Differential recruitment of CD63 and Rab7-interacting-lysosomal-protein to phagosomes containing *Mycobacterium tuberculosis* in macrophages. Microbiol Immunol 54: 170-174.

S23. Cardoso CM, Jordao L, Vieira OV (2010) Rab10 regulates phagosome maturation and its overexpression rescues Mycobacterium-containing phagosomes maturation. Traffic 11: 221-235.

S24. Sun J, Deghmane AE, Soualhine H, Hong T, Bucci C, Solodkin A, Hmama Z (2007) *Mycobacterium bovis* BCG disrupts the interaction of Rab7 with RILP contributing to inhibition of phagosome maturation. J Leukoc Biol 82: 1437-1445.

S25. Kyei GB, Vergne I, Chua J, Roberts E, Harris J, Junutula JR, Deretic V (2006) Rab14 is critical for maintenance of *Mycobacterium tuberculosis* phagosome maturation arrest. EMBO J 25: 5250-5259.

S26. Harrison RE, Brumell JH, Khandani A, Bucci C, Scott CC, Jiang X, Finlay BB, Grinstein S (2004) Salmonella impairs RILP recruitment to Rab7 during maturation of invasion vacuoles. Mol Biol Cell 15: 3146-3154.

S27. Ohlson MB, Huang Z, Alto NM, Blanc MP, Dixon JE, Chai J, Miller SI (2008) Structure and function of Salmonella SifA indicate that its interactions with SKIP, SseJ, and RhoA family GTPases induce endosomal tubulation. Cell Host Microbe 4: 434-446.

S28. Jackson LK, Nawabi P, Hentea C, Roark EA, Haldar K (2008) The Salmonella virulence protein SifA is a G protein antagonist. Proc Natl Acad Sci U S A 105: 14141-14146.

S29. Fratti RA, Chua J, Vergne I, Deretic V (2003) *Mycobacterium tuberculosis* glycosylated phosphatidylinositol causes phagosome maturation arrest. Proc Natl Acad Sci U S A 100: 5437-5442.

S30. Kang PB, Azad AK, Torrelles JB, Kaufman TM, Beharka A, Tibesar E, DesJardin LE, Schlesinger LS (2005) The human macrophage mannose receptor directs *Mycobacterium tuberculosis* lipoarabinomannan-mediated phagosome biogenesis. J Exp Med 202: 987-999.

S31. Horwitz MA (1983) The Legionnaires' disease bacterium (*Legionella pneumophila*) inhibits phagosome-lysosome fusion in human monocytes. J Exp Med 158: 2108-2126.

S32. Sturgill-Koszycki S, Swanson MS (2000) *Legionella pneumophila* replication vacuoles mature into acidic, endocytic organelles. J Exp Med 192: 1261-1272.

S33. Chang KP, Dwyer DM (1976) Multiplication of a human parasite (*Leishmania donovani*) in phagolysosomes of hamster macrophages in vitro. Science 193: 678-680.

S34. Scianimanico S, Desrosiers M, Dermine JF, Meresse S, Descoteaux A, Desjardins M (1999) Impaired recruitment of the small GTPase Rab7 correlates with the inhibition of phagosome maturation by *Leishmania donovani* promastigotes. Cell Microbiol 1: 19-32.

S35. Spath GF, Garraway LA, Turco SJ, Beverley SM (2003) The role(s) of lipophosphoglycan (LPG) in the establishment of *Leishmania major* infections in mammalian hosts. Proc Natl Acad Sci U S A 100: 9536-9541.

S36. Howe D, Mallavia LP (2000) *Coxiella burnetii* exhibits morphological change and delays phagolysosomal fusion after internalization by J774A.1 cells. Infect Immun 68: 3815-3821.

S37. Akporiaye ET, Rowatt JD, Aragon AA, Baca OG (1983) Lysosomal response of a murine macrophage-like cell line persistently infected with *Coxiella burnetii.* Infect Immun 40: 1155-1162.

S38. Heinzen RA, Scidmore MA, Rockey DD, Hackstadt T (1996) Differential interaction with endocytic and exocytic pathways distinguish parasitophorous vacuoles of *Coxiella burnetii* and *Chlamydia trachomatis*. Infect Immun 64: 796-809.

S39. McConville MJ, Blackwell JM (1991) Developmental changes in the glycosylated phosphatidylinositols of *Leishmania donovani*. Characterization of the promastigote and amastigote glycolipids. J Biol Chem 266: 15170-15179.

S40. Amer A, Franchi L, Kanneganti TD, Body-Malapel M, Ozoren N, Brady G, Meshinchi S, Jagirdar R, Gewirtz A, Akira S, Nunez G (2006) Regulation of *Legionella* phagosome maturation and infection through flagellin and host Ipaf. J Biol Chem 281: 35217-35223.

S41. Fernandez-Moreira E, Helbig JH, Swanson MS (2006) Membrane vesicles shed by *Legionella pneumophila* inhibit fusion of phagosomes with lysosomes. Infect Immun 74: 3285-3295.

S42. Schaible UE, Schlesinger PH, Steinberg TH, Mangel WF, Kobayashi T, Russell DG (1999) Parasitophorous vacuoles of *Leishmania mexicana* acquire macromolecules from the host cell cytosol via two independent routes. J Cell Sci 112 ( Pt 5): 681-693.

S43. Beron W, Gutierrez MG, Rabinovitch M, Colombo MI (2002) *Coxiella burnetii* localizes in a Rab7-labeled compartment with autophagic characteristics. Infect Immun 70: 5816-5821.

S44. Gutierrez MG, Vazquez CL, Munafo DB, Zoppino FC, Beron W, Rabinovitch M, Colombo MI (2005) Autophagy induction favours the generation and maturation of the *Coxiella*-replicative vacuoles. Cell Microbiol 7: 981-993.

S45. Hackstadt T, Williams JC (1981) Biochemical stratagem for obligate parasitism of eukaryotic cells by *Coxiella burnetii*. Proc Natl Acad Sci U S A 78: 3240-3244.

S46. Yasir M, Pachikara ND, Bao X, Pan Z, Fan H (2011) Regulation of Chlamydial Infection by Host Autophagy and Vacuolar ATPase-Bearing Organelles. Infect Immun 79: 4019-4028.

S47. Pizarro-Cerda J, Moreno E, Sanguedolce V, Mege JL, Gorvel JP (1998) Virulent *Brucella abortus* prevents lysosome fusion and is distributed within autophagosome-like compartments. Infect Immun 66: 2387-2392.

S48. Moreau K, Lacas-Gervais S, Fujita N, Sebbane F, Yoshimori T, Simonet M, Lafont F (2010) Autophagosomes can support *Yersinia pseudotuberculosis* replication in macrophages. Cell Microbiol 12: 1108-1123.

S49. Pujol C, Klein KA, Romanov GA, Palmer LE, Cirota C, Zhao Z, Bliska JB (2009) *Yersinia pestis* can reside in autophagosomes and avoid xenophagy in murine macrophages by preventing vacuole acidification. Infect Immun 77: 2251-2261.

S50. Kocks C, Hellio R, Gounon P, Ohayon H, Cossart P (1993) Polarized distribution of *Listeria monocytogenes* surface protein ActA at the site of directional actin assembly. J Cell Sci 105 ( Pt 3): 699-710.

S51. Welch MD, Iwamatsu A, Mitchison TJ (1997) Actin polymerization is induced by Arp2/3 protein complex at the surface of *Listeria monocytogenes*. Nature 385: 265-269.

S52. Egile C, Loisel TP, Laurent V, Li R, Pantaloni D, Sansonetti PJ, Carlier MF (1999) Activation of the CDC42 effector N-WASP by the *Shigella flexneri* IcsA protein promotes actin nucleation by Arp2/3 complex and bacterial actin-based motility. J Cell Biol 146: 1319-1332.

S53. Leung Y, Ally S, Goldberg MB (2008) Bacterial actin assembly requires toca-1 to relieve N-wasp autoinhibition. Cell Host Microbe 3: 39-47.

S54. Andrade LO, Andrews NW (2004) Lysosomal fusion is essential for the retention of *Trypanosoma cruzi* inside host cells. J Exp Med 200: 1135-1143.

S55. Andrews NW, Whitlow MB (1989) Secretion by *Trypanosoma cruzi* of a hemolysin active at low pH. Mol Biochem Parasitol 33: 249-256.

S56. Jabado N, Jankowski A, Dougaparsad S, Picard V, Grinstein S, Gros P (2000) Natural resistance to intracellular infections: natural resistance-associated macrophage protein 1 (Nramp1) functions as a pH-dependent manganese transporter at the phagosomal membrane. J Exp Med 192: 1237-1248.

S57. Champion OL, Karlyshev A, Cooper IA, Ford DC, Wren BW, Duffield M, Oyston PC, Titball RW (2011) *Yersinia pseudotuberculosis* mntH functions in intracellular manganese accumulation, which is essential for virulence and survival in cells expressing functional Nramp1. Microbiology 157: 1115-1122.

S58. Anderson ES, Paulley JT, Gaines JM, Valderas MW, Martin DW, Menscher E, Brown TD, Burns CS, Roop RM (2009) The manganese transporter MntH is a critical virulence determinant for *Brucella abortus* 2308 in experimentally infected mice. Infect Immun 77: 3466-3474.

S59. Patel N, Singh SB, Basu SK, Mukhopadhyay A (2008) *Leishmania* requires Rab7-mediated degradation of endocytosed hemoglobin for their growth. Proc Natl Acad Sci U S A 105: 3980-3985.

S60. Britigan BE, Lewis TS, McCormick ML, Wilson ME (1998) Evidence for the existence of a surface receptor for ferriclactoferrin and ferrictransferrin associated with the plasma membrane of the protozoan parasite *Leishmania donovani*. Adv Exp Med Biol 443: 135-140.

S61. Das NK, Biswas S, Solanki S, Mukhopadhyay CK (2009) *Leishmania donovani* depletes labile iron pool to exploit iron uptake capacity of macrophage for its intracellular growth. Cell Microbiol 11: 83-94.

S62. Crouch ML, Castor M, Karlinsey JE, Kalhorn T, Fang FC (2008) Biosynthesis and IroC-dependent export of the siderophore salmochelin are essential for virulence of *Salmonella enterica* serovar Typhimurium. Mol Microbiol 67: 971-983.

S63. Luo M, Lin H, Fischbach MA, Liu DR, Walsh CT, Groves JT (2006) Enzymatic tailoring of enterobactin alters membrane partitioning and iron acquisition. ACS Chem Biol 1: 29-32.

S64. Fetherston JD, Kirillina O, Bobrov AG, Paulley JT, Perry RD (2010) The yersiniabactin transport system is critical for the pathogenesis of bubonic and pneumonic plague. Infect Immun 78: 2045-2052.

S65. Tullius MV, Harmston CA, Owens CP, Chim N, Morse RP, McMath LM, Iniguez A, Kimmey JM, Sawaya MR, Whitelegge JP, Horwitz MA, Goulding CW (2011) Discovery and characterization of a unique mycobacterial heme acquisition system. Proc Natl Acad Sci U S A 108: 5051-5056.

S66. Munoz-Elias EJ, Upton AM, Cherian J, McKinney JD (2006) Role of the methylcitrate cycle in *Mycobacterium tuberculosis* metabolism, intracellular growth, and virulence. Mol Microbiol 60: 1109-1122.

S67. Van der GR, Yam K, Heuser T, Wilbrink MH, Hara H, Anderton MC, Sim E, Dijkhuizen L, Davies JE, Mohn WW, Eltis LD (2007) A gene cluster encoding cholesterol catabolism in a soil actinomycete provides insight into *Mycobacterium tuberculosis* survival in macrophages. Proc Natl Acad Sci U S A 104: 1947-1952.

S68. Bowden SD, Rowley G, Hinton JC, Thompson A (2009) Glucose and glycolysis are required for the successful infection of macrophages and mice by *Salmonella enterica* serovar typhimurium. Infect Immun 77: 3117-3126.

S69. Mercado-Lubo R, Leatham MP, Conway T, Cohen PS (2009) *Salmonella enterica* serovar Typhimurium mutants unable to convert malate to pyruvate and oxaloacetate are avirulent and immunogenic in BALB/c mice. Infect Immun 77: 1397-1405.

S70. Romano PS, Gutierrez MG, Beron W, Rabinovitch M, Colombo MI (2007) The autophagic pathway is actively modulated by phase II *Coxiella burnetii* to efficiently replicate in the host cell. Cell Microbiol 9: 891-909.

S71. Gallois A, Klein JR, Allen LA, Jones BD, Nauseef WM (2001) Salmonella pathogenicity island 2-encoded type III secretion system mediates exclusion of NADPH oxidase assembly from the phagosomal membrane. J Immunol 166: 5741-5748.

S72. Lodge R, Diallo TO, Descoteaux A (2006) *Leishmania donovani* lipophosphoglycan blocks NADPH oxidase assembly at the phagosome membrane. Cell Microbiol 8: 1922-1931.

S73. Brehm K, Haas A, Goebel W, Kreft J (1992) A gene encoding a superoxide dismutase of the facultative intracellular bacterium *Listeria monocytogenes*. Gene 118: 121-125.

S74. Roggenkamp A, Bittner T, Leitritz L, Sing A, Heesemann J (1997) Contribution of the Mn-cofactored superoxide dismutase (SodA) to the virulence of *Yersinia enterocolitica* serotype O8. Infect Immun 65: 4705-4710.

S75. Bakshi CS, Malik M, Regan K, Melendez JA, Metzger DW, Pavlov VM, Sellati TJ (2006) Superoxide dismutase B gene (sodB)-deficient mutants of *Francisella tularensis* demonstrate hypersensitivity to oxidative stress and attenuated virulence. J Bacteriol 188: 6443-6448.

S76. Braunstein M, Espinosa BJ, Chan J, Belisle JT, Jacobs WR, Jr. (2003) SecA2 functions in the secretion of superoxide dismutase A and in the virulence of *Mycobacterium tuberculosis*. Mol Microbiol 48: 453-464.

S77. Wu CH, Tsai-Wu JJ, Huang YT, Lin CY, Lioua GG, Lee FJ (1998) Identification and subcellular localization of a novel Cu,Zn superoxide dismutase of *Mycobacterium tuberculosis*. FEBS Lett 439: 192-196.

S78. Burrows TW, Farrell JM, Gillett WA (1964) The catalase activities of *Pasteurella pestis* and other bacteria. BR J Exp Pathol 45: 579-588.

S79. Garcia E, Nedialkov YA, Elliott J, Motin VL, Brubaker RR (1999) Molecular characterization of KatY (antigen 5), a thermoregulated chromosomally encoded catalase-peroxidase of *Yersinia pestis*. J Bacteriol 181: 3114-3122.

S80. McLean S, Bowman LA, Poole RK (2010) KatG from *Salmonella typhimurium* is a peroxynitritase. FEBS Lett 584: 1628-1632.

S81. Pym AS, Domenech P, Honore N, Song J, Deretic V, Cole ST (2001) Regulation of catalase-peroxidase (KatG) expression, isoniazid sensitivity and virulence by furA of *Mycobacterium tuberculosis*. Mol Microbiol 40: 879-889.

S82. Robbe-Saule V, Coynault C, Ibanez-Ruiz M, Hermant D, Norel F (2001) Identification of a non-haem catalase in Salmonella and its regulation by RpoS (sigmaS). Mol Microbiol 39: 1533-1545.

S83. Alvarez MN, Peluffo G, Piacenza L, Radi R (2011) Intraphagosomal peroxynitrite as a macrophage-derived cytotoxin against internalized *Trypanosoma cruzi*: consequences for oxidative killing and role of microbial peroxiredoxins in infectivity. J Biol Chem 286: 6627-6640.

S84. Couture M, Yeh SR, Wittenberg BA, Wittenberg JB, Ouellet Y, Rousseau DL, Guertin M (1999) A cooperative oxygen-binding hemoglobin from *Mycobacterium tuberculosis*. Proc Natl Acad Sci U S A 96: 11223-11228.

S85. Ouellet H, Ouellet Y, Richard C, Labarre M, Wittenberg B, Wittenberg J, Guertin M (2002) Truncated hemoglobin HbN protects *Mycobacterium bovis* from nitric oxide. Proc Natl Acad Sci U S A 99: 5902-5907.

S86. Fabozzi G, Ascenzi P, Renzi SD, Visca P (2006) Truncated hemoglobin GlbO from *Mycobacterium leprae* alleviates nitric oxide toxicity. Microb Pathog 40: 211-220.

S87. Mills PC, Rowley G, Spiro S, Hinton JC, Richardson DJ (2008) A combination of cytochrome c nitrite reductase (NrfA) and flavorubredoxin (NorV) protects *Salmonella enterica* serovar Typhimurium against killing by NO in anoxic environments. Microbiology 154: 1218-1228.

S88. Keller P, Schaumburg F, Fischer SF, Hacker G, Gross U, Luder CG (2006) Direct inhibition of cytochrome c-induced caspase activation in vitro by *Toxoplasma gondii* reveals novel mechanisms of interference with host cell apoptosis. FEMS Microbiol Lett 258: 312-319.

S89. Hashimoto M, Nakajima-Shimada J, Aoki T (2005) *Trypanosoma cruzi* posttranscriptionally up-regulates and exploits cellular FLIP for inhibition of death-inducing signal. Mol Biol Cell 16: 3521-3528.

S90. Goebel S, Gross U, Luder CG (2001) Inhibition of host cell apoptosis by *Toxoplasma gondii* is accompanied by reduced activation of the caspase cascade and alterations of poly(ADP-ribose) polymerase expression. J Cell Sci 114: 3495-3505.

S91. Akarid K, Arnoult D, Micic-Polianski J, Sif J, Estaquier J, Ameisen JC (2004) *Leishmania major*-mediated prevention of programmed cell death induction in infected macrophages is associated with the repression of mitochondrial release of cytochrome c. J Leukoc Biol 76: 95-103.

S92. Zhang JZ, Sinha M, Luxon BA, Yu XJ (2004) Survival strategy of obligately intracellular *Ehrlichia chaffeensis*: novel modulation of immune response and host cell cycles. Infect Immun 72: 498-507.

S93. Mansell A, Braun L, Cossart P, O'Neill LA (2000) A novel function of InIB from *Listeria monocytogenes*: activation of NF-kappaB in J774 macrophages. Cell Microbiol 2: 127-136.

S94. bu-Zant A, Santic M, Molmeret M, Jones S, Helbig J, Abu KY (2005) Incomplete activation of macrophage apoptosis during intracellular replication of *Legionella pneumophila.* Infect Immun 73: 5339-5349.

S95. Laguna RK, Creasey EA, Li Z, Valtz N, Isberg RR (2006) A *Legionella pneumophila*-translocated substrate that is required for growth within macrophages and protection from host cell death. Proc Natl Acad Sci U S A 103: 18745-18750.

S96. Kamir D, Zierow S, Leng L, Cho Y, Diaz Y, Griffith J, McDonald C, Merk M, Mitchell RA, Trent J, Chen Y, Kwong YK, Xiong H, Vermeire J, Cappello M, Mahon-Pratt D, Walker J, Bernhagen J, Lolis E, Bucala R (2008) A *Leishmania* ortholog of macrophage migration inhibitory factor modulates host macrophage responses. J Immunol 180: 8250-8261.

S97. Hwang IY, Quan JH, Ahn MH, Ahmed HA, Cha GH, Shin DW, Lee YH (2010) *Toxoplasma gondii* infection inhibits the mitochondrial apoptosis through induction of Bcl-2 and HSP70. Parasitol Res 107: 1313-1321.

S98. Schotte P, Denecker G, Van Den BA, Vandenabeele P, Cornelis GR, Beyaert R (2004) Targeting Rac1 by the *Yersinia* effector protein YopE inhibits caspase-1-mediated maturation and release of interleukin-1β. J Biol Chem 279: 25134-25142.

S99. Monack DM, Mecsas J, Bouley D, Falkow S (1998) *Yersinia*-induced apoptosis in vivo aids in the establishment of a systemic infection of mice. J Exp Med 188: 2127-2137.

S100. Keane J, Remold HG, Kornfeld H (2000) Virulent *Mycobacterium tuberculosis* strains evade apoptosis of infected alveolar macrophages. J Immunol 164: 2016-2020.

S101. Bannai H, Nishikawa Y, Matsuo T, Kawase O, Watanabe J, Sugimoto C, Xuan X (2008) Programmed Cell Death 5 from *Toxoplasma gondii*: a secreted molecule that exerts a pro-apoptotic effect on host cells. Mol Biochem Parasitol 159: 112-120.

S102. Das G, Vohra H, Rao K, Saha B, Mishra GC (1999) *Leishmania donovani* infection of a susceptible host results in CD4+ T-cell apoptosis and decreased Th1 cytokine production. Scand J Immunol 49: 307-310.

S103. Kremer L, Estaquier J, Wolowczuk I, Biet F, Ameisen JC, Locht C (2000) Ineffective cellular immune response associated with T-cell apoptosis in susceptible *Mycobacterium bovis* BCG-infected mice. Infect Immun 68: 4264-4273.

S104. Hoette TM, Clifton MC, Zawadzka AM, Holmes MA, Strong RK, Raymond KN (2011) Immune Interference in *Mycobacterium tuberculosis* Intracellular Iron Acquisition through Siderocalin Recognition of Carboxymycobactins. ACS Chem Biol .
